# Health of the British Army and Navy Compared Together

**Published:** 1862-08

**Authors:** 


### HEALTH OF THE BRITISH ARMY AND NAVY COMPARED TOGETHER.

Dr. Milroy read a paper on this subject before the Social Science Congress at its recent meeting. It contained the following startling facts in reference to the two services: In the army, in 1859, in the United Kingdom, the annual death-rate was 8.4 in 1000; in the Mediterranean it was 13; in the West Indies, 17; in the Australian colonies, 10; and in Ceylon and China, 47.1. The death-rate in the navy was as follows for the year 1858: On the home station, 9.6; Mediterranean, 11.1; West Indian, 20.8; Australian, 7.9; and East Indian and China, 62.5. The daily sick-rate in the army was as follows: United Kingdom, 50 in 1000; Mediterranean 48; West Indies, 53.5; Australian colonies, 27; Ceylon and China, 99.5. The daily sick-rate in the navy was: Home station, 53.5; Mediterranean, 48.7; West Indian, 66.6; Australian colonies, 42; and East Indies and China, 92.

A discussion ensued upon this paper in which Mr. J. N. Redcliffe said it was worthy of notice that Lord Clarence Paget in moving the navy estimates, had stated that a large amount of the mortality and sickness in ships was believed to be traceable to the want of proper ventilation between decks; but was not aware that the ventilation of iron-plated ships had been brought under the notice of the parliament committee which had been appointed to consider the subject. The iron-plating of ships shut out the means of ventilation, and the temperature between the decks of the *Warrior* was much higher than in any of the other war ships of the navy now in use. He understood that the Government had directed that seventy tons of solid brickwork should be placed on the bottom of the ship to keep out the bilge-water, but whether or not it was to be cemented he did not know. At the same time he must say that, when this brick-work should become impregnated with bilge-water, the evils would be greatly aggravated. Therefore, what the condition of the iron-plated ships now building would be could be readily conceived, and he thought it was urgently required that the attention of the Government should be drawn to the ventilation of the *Warrior*, *Black Prince*, *Defence*, and the other iron-plated ships now building, or the mortality might prove much greater than it was at the present time.—*Dublin Medical Journal*, June 25, 1862.

